# Herbivore space use influences coral reef recovery

**DOI:** 10.1098/rsos.160262

**Published:** 2016-06-29

**Authors:** Yoan Eynaud, Dylan E. McNamara, Stuart A. Sandin

**Affiliations:** 1 Center for Marine Biodiversity and Conservation, Scripps Institution of Oceanography, 9500 Gilman Drive, La Jolla, CA 92093-0202, USA; 2 Department of Physics and Physical Oceanography/Center for Marine Science, University of North Carolina-Wilmington, 601 South College Road, Wilmington, NC 28403, USA

**Keywords:** herbivore, benthic communities, recovery, behaviour, space use, modelling

## Abstract

Herbivores play an important role in marine communities. On coral reefs, the diversity and unique feeding behaviours found within this functional group can have a comparably diverse set of impacts in structuring the benthic community. Here, using a spatially explicit model of herbivore foraging, we explore how the spatial pattern of grazing behaviours impacts the recovery of a reef ecosystem, considering movements at two temporal scales—short term (e.g. daily foraging patterns) and longer term (e.g. monthly movements across the landscape). Model simulations suggest that *more spatially constrained herbivores* are more effective at conferring recovery capability by providing a favourable environment to coral recruitment and growth. Results also show that the composition of food available to the herbivore community is linked directly to the pattern of space use by herbivores. To date, most studies of variability among the impacts of herbivore species have considered the diversity of feeding modes and mouthparts. Our work provides a complementary view of spatial patterns of foraging, revealing that variation in movement behaviours alone can affect patterns of benthic change, and thus broadens our view of realized links between herbivore diversity and reef recovery.

## 1. Introduction

The benthic environment of a coral reef is spatially and taxonomically heterogeneous, with significant variation both within and across habitat zones (e.g. depth, hydrodynamic regimes). The drivers of such spatial and taxonomic heterogeneity are difficult to identify completely. Nevertheless, it is likely that competition for space plays a central role in controlling spatial patterning and diversity [1]. Many depictions of change of the reef benthos are reported in per cent cover of functional groups through time, thereby summarizing the mean coverage of a given taxon and ignoring spatial configurations. However, in order to understand more fully the mechanisms of benthic change, it is critical to track spatially explicit patterns of change among reef organisms. Indeed, most benthic patterns are the product of competitive battles for limited space on the bottom.

Herbivores have an important role in affecting the spatial patterning of reef environments. Herbivores can consume algae and reduce the competitive advantage of algae versus corals, therefore, in many examples, the ratio of coral to algal cover is affected, if not determined, by the activities of herbivores [2,3]. In the presence of abundant herbivores, corals can outcompete algae for space. In the presence of few herbivores (e.g. when herbivores are removed by fishing activities), algae can outcompete corals for space through several mechanisms (e.g. pathogens [4], negative allelopathy [5], shading [6] and microbial fertilization [7]). Moreover, the spatial impact of herbivory can vary significantly, from the spatially static impact made by sea urchins or site-faithful territorial fishes to the spatially varied impact made by a school of roving fish. Thus, the impact that herbivores have in altering the outcome of coral–algal interactions is spatially heterogeneous [8] and when considering the roles played by herbivores, it is crucial to treat their patterns of space use. Many examples of how space use among herbivores in other ecosystems influences the evolution of sessile community patterns exist [9,10]. Given the wide variety of herbivorous species and feeding modes on coral reefs, there is reason to expect comparable linkages between feeding behaviour and spatial patterning of the benthos.

Recently, Sandin & McNamara [11] used a cellular automata model to explore the importance of variation in one aspect of herbivore movements on the benthic dynamics of coral reefs. The model simulations revealed that when short-timescale foraging patterns (i.e. grazing movement on daily scales) were spatially constrained (mimicking the behaviour of herbivorous sea urchins), herbivory was effective at creating sufficient open space free of fleshy algae to facilitate settlement of corals and subsequent recruitment to adult populations. By contrast, when short-timescale foraging patterns were not spatially constrained (mimicking the behaviour of a generalized groups of herbivorous fishes), herbivory was ineffective at facilitating coral recruitment except at high densities of herbivores. These model simulations treated only one dimension of spatial patterning of herbivory; most herbivorous taxa on a coral reef, however, also show long-timescale (monthly) patterns of movement across the reef landscape.

Further, Sandin & McNamara [11] focused solely on how the attractor (or putative ‘steady state’)—the coral-dominated state or the algae-dominated state—reached by the system is impacted by the two end-member possibilities of short-term foraging patterns. Such an approach is valuable for considering long-term development of reef benthic configuration, but is insufficient for exploring rates of change of the benthic environment. In many applications, both for basic understanding and for reef management, it is helpful to understand the influence of a range of herbivore space use on the post-disturbance recovery rate of coral reefs. Our interest here is not in understanding how steady-state dynamics are influenced by changes in herbivory intensity but rather how the herbivore space use influences transient dynamics as reefs evolve towards a coral-dominated state.

In this study, we consider how a spectrum of herbivory patterns can affect the outcome of competition for space among benthic reef organisms. More precisely, we aim to identify the range of grazing behaviours that appear effective at conferring recovery capabilities to the reef. Thus, by comparing how the reef recovered from a given perturbation after a fixed time, over a range of herbivory behaviour, we broadly characterize recovery dynamics of the coral reef.

This work is organized into two sections. (i) We first define a generalizable representation of herbivore-grazing behaviour that focuses on explicit patterns of space use among the herbivorous guild. Such a model allows us to represent a wide range of grazing behaviours using a simple formalism. (ii) Using the approach defined above, we explore how recovery is impacted by the spatial behaviours of grazers.

## 2. Material and methods

A spatially explicit model of the coral reef benthos was used as the context within which to explore benthic dynamics over a range of differing space use by herbivores. We extend the existing cellular automata model that has been used to investigate benthic behaviour under stereotypical patterns of herbivory (i.e. strictly site-attached sea urchins versus spatially unconstrained ‘fish’) and coral growth morphology (i.e. branching versus massive) [11]. The extended model is used to explore a spectrum of herbivore movement patterns by implementing a novel representation of herbivore impact on the simulated benthic community, including both short-timescale (daily) and long-timescale (monthly) patterns of movement of the herbivorous guild.

### 2.1. Model domain

The model domain uses periodic boundary conditions and comprises square cells. Each cell is occupied by one of four benthic types: stony corals, primary algal competitor, secondary algal competitor or crustose coralline algae/empty space. These four benthic types were defined to capture spatial dynamics of the dominant benthic reef organisms, particularly patterns of recruitment, mortality and competition for space. Stony corals have low recruitment rates, but adults (here defined as colonies larger than 900 cm^2^, see table 1) are long lived and are dominant competitors for space [12]. The secondary algal competitors have high recruitment rates relative to corals but are poor competitors for space [13]. The primary algal competitors are intermediate competitors for space only inferior to adult corals in their competitive abilities. Each of these three types can grow clonally (or vegetatively), laterally expanding to adjacent cells. Finally, crustose coralline algae are foundational species on the reef, living beneath other benthic types. Here we make the assumption that the crustose coralline algae group is not an active competitor for space, ignoring their possible competitive effects towards coral and other benthic organisms.

**Table 1.** Basic parameter definition for the simulation model. Note: the contiguous threshold for coral term, *C*_th_, refers to the size threshold at which corals are considered adults and start to outcompete the primary algal competitor. For more information see [11].

parameters	interpretation	value	unit
*P*_Cr_	probability of recruitment for C	0.01	yr^−1^
*P*_SACr_	probability of recruitment for the secondary algal competitor	0.80	yr^−1^
*G*_C_	growth term for C	0.01	m yr^−1^
*G*_SAC_	growth term for the secondary algal competitor	1	m yr^−1^
*G*_PAC_	growth term for the primary algal competitor	0.5	m yr^−1^
*P*_S-PAC_	probability of succession from the secondary to the primary algal competitor	0.33	yr^−1^
*P*_Cd_	probability of mortality of C	0.15	yr^−1^
*C*_th_	contiguous threshold for coral	900	cm^2^
*S*_R_	size of recruit (for every state)	100	cm^2^

**Figure 1.**
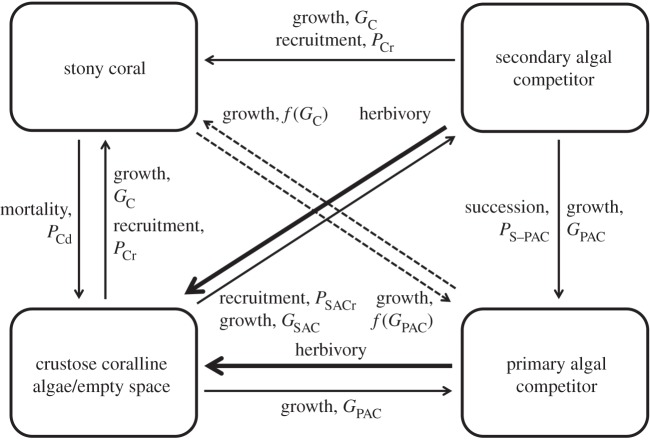
Flow chart of coral reef benthic interactions. Transitions among the four functional groups are due to recruitment, mortality, herbivory, growth and succession. Important parameters are included in each link and represent transition probabilities. State-dependent competitive dynamics are indicated by dashed lines. Parameters are defined in table 1.

Transitions among the four functional groups are due to recruitment, mortality, herbivory, growth and succession. Crustose coralline algae/empty space cells may transition into stony coral or the secondary algal competitor cells through processes of recruitment or growth of an adjacent cell or may transition into primary algal competitor cells via the growth from an adjacent cell. Secondary algal competitor cells may transition into stony coral cells through recruitment or growth of an adjacent cell or into primary algal competitor cells through succession and growth of an adjacent cell. Primary algal competitor cells may transition into stony coral cells through growth of an adjacent stony coral cell. Once grazed, any algal cell (secondary and primary competitor) will transition towards a crustose coralline algae/empty cell. Finally, stony coral cells may transition towards a crustose coralline algae/empty cell due to mortality or may transition towards the primary algal competitor cell if overgrown. A schematic of modelled species interactions along with a table of basal parameter values are provided in figure 1 and table 1, respectively. For further details regarding the parametrization and construction of the benthic dynamic model, refer to Sandin & McNamara [11].

In most cases, one could refer to the primary and secondary algal competitors using the following ecological guilds—primary: macroalgae and thick turf algae, and secondary: turf algae. Indeed, turf is usually described as a complex assemblage of pioneering and less competitive algae from which, following succession, more competitive algae, notably thick, complex turf assemblages and macroalgae, emerge [14–16]. However, we emphasize that our naming convention explicitly refers to the relative capabilities of erect algae to compete for space as our goal is to differentiate types of algae that can be grazed by herbivores and potentially outcompete stony corals (i.e. primary competitors) from types that can be grazed by herbivores but are much less spatially competitive (i.e. secondary competitors).

Model results presented below use a grid cell size of 10 × 10 cm (100 cm^2^) within a 5 × 5 m (25 m^2^) domain and time steps of 2 × 10^−2^ year, approximately one week. The novel formulation of herbivores as well as the definition and characterization of transient dynamics are introduced below.

### 2.2. Herbivore model

Functional diversity in spatial behaviour is mainly a matter of timescale; for example, whereas some fish can cover nearly a 100 m^2^ in 1 h, some sea urchins can only cover a few square meters in several days. As our goal is to focus on the dynamics of reef recovery, which occur over many weeks to years, it is intractable and dynamically inconsistent to reduce grazing behaviour to individual species and individual feeding mechanisms [17]. Furthermore, a structurally complex grazing model built on individuals would rely on numerous, summed parametrizations of movement behaviours and feeding mechanics. This would make it very difficult to understand links between results and assumptions made in the parametrizations [18]. Thus, we chose to represent the impact of the entire herbivore community on the benthos (parsimoniously summarized based upon spatial foraging patterns) rather than the context-specific and parameter-intensive representation of individual herbivore-grazing action combined across the assemblage. Our represented herbivore behaviour is to be seen as an abstracted version of detailed short-timescale dynamics appropriate to the coral–algal competition scale of interest [17], a so-called ‘rain of bites’ that lands on the benthic community [19].

Grazing activity is characterized by the disappearance of 100 cm^2^ algal cells and a resulting transition towards a crustose coralline algal/empty space cell. We thus model herbivory by means of collective scraping and grazing (as defined in [20] and references therein) and ignore the detailed feeding mechanisms that could lead to conversion of the primary algal competitor into the secondary algal competitor. Also, the probability for an algal cell to be grazed in this formulation is independent of its type (i.e. primary and secondary algal competitors, if encountered, are equally likely to be consumed). While such an abstraction is common in coral reef benthic modelling [21–23], we have studied its consequences relative to more specialized feeding models. More precisely, we have explored a wide range of foraging biases of the herbivore assemblage, from strong preference for primary algal competitors to a strong preference for secondary algal competitors. The obtained results have shown that the dynamical effects of spatial patterns of herbivory are prominent in affecting patterns of reef recovery with only a weak effect of foraging bias between primary and secondary algal competitors (see electronic supplementary material, appendix S1). As we have revealed that foraging preference by herbivores does not alter strongly the model outcomes when exploring the influence of spatial patterning of herbivores, we present model results for the simpler model below.

The distribution of herbivore movements defines the spatial heterogeneity of herbivore impact across a landscape and can be divided into two types, depending on the timescale and the frequency at which movement takes place. On one hand, high-frequency movements (hours to days), mainly motivated by short-term and context-specific foraging decisions, are represented by the disappearance of several algal cells following a given spatial distribution centred over a given cell within the grid. On the other hand, low-frequency movements (weeks to years), mainly motivated by longer term homing and patterns of landscape use, are represented by the movement of the centre of the grazed cells spatial distribution. We simulate those two types of movement separately using two probability distributions defined over the represented area as follows: (i) the grazing node, representing movements motivated by foraging; (ii) the homing node, representing movements motivated by homing purposes (for homing species) and/or the search of new foraging areas (for roving and transient species). Both nodes have the same centre positioned on a given cell within the grid, i.e. the node centre.

**Figure 2.**
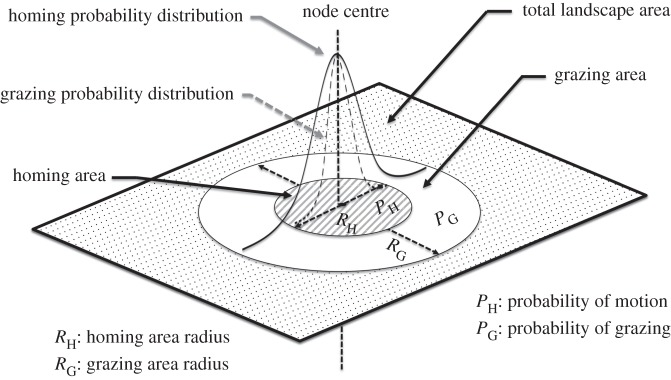
A graphical representation of generalized spatial patterns of herbivory. A grazing node is defined by its central position on the two-dimensional domain, called here the node centre. A given algal cell may be grazed following a given probability distribution within a circle of radius *R*_G_ around the node centre. A given cell may become the new node centre with a given probability distribution within a circle of radius *R*_H_ around the previous position of the node centre. *R*_G_ can be larger than *R*_H_ and vice versa. Here two probability distributions are applied over both the homing and grazing node. The maximum number of bites per week is denoted *B*_M_.

In the absence of detailed field data on herbivore spatial foraging behaviour, we use two classic probability distributions for each node, a two-dimensional Gaussian distribution and a uniform distribution (see electronic supplementary material, appendix S2 for details). The topological structure of our modelled benthos, a torus, forbids us to use a Gaussian distribution, *sensu stricto.* Indeed, a Gaussian distribution is defined on an infinite domain, whereas a torus is a finite domain. Thus, we use circularly truncated distributions for both the homing and grazing node. Therefore, both our grazing and homing node can be characterized using only one parameter: the radius of the circle upon which they are defined, respectively, *R*_G_ and *R*_H_. To allow direct comparison, we use the same truncation procedure when implementing a uniform distribution. Again, grazing in the model is simulated by the transition of an algal cell to a crustose coralline algae/empty space cell. Therefore, we can constrain herbivore impact by controlling the maximum number of cells bitten per week. We, therefore, represent the space use of herbivores with only three parameters: the grazing node size (a circle of size $\pi \times R_{G}^{2}$), the homing node size (a circle of size $\pi \times R_{H}^{2}$), and the maximum number of bites, or unitary removal of 100 cm^2^ algal cells (*B*_M_) (figure 2). In the model, at each time step, a maximum number of algal cells *B*_M_ can be grazed within a circular area centred on the node centre and of size $\pi \times R_{G}^{2}$ following a given probability distribution (Gaussian or uniform). The node centre can be relocated, at each time step, in a cell contained within a circular area centred on its position and of size $\pi \times R_{H}^{2}$, again following a given probability distribution (Gaussian or uniform).

The relative simplicity of the three-parameter herbivore representation highlights our interest in understanding the evolution of coral reef benthic structure over a wide range of space use patterns by herbivores rather than simulating the dynamics of a specific reef location. Three examples illustrate the model's capability of representing a broad spectrum of herbivory: (i) a relatively small grazing node size associated with a very small (if not null) homing node size will match, for example, the behaviour exhibited by a herbivore community dominated by urchins practising homing or territorial herbivorous pomacentrids [24]; (ii) a relatively small grazing node size associated with an intermediate homing node size will match, for example, the behaviour exhibited by a herbivore community dominated by site-faithful surgeonfishes; (iii) a relatively large grazing node size associated with a large homing node size will match, for example, the behaviour exhibited by a herbivore community dominated by large, roving parrotfishes.

### 2.3. Numerical analyses

In order to explore the role of herbivore spatial patterning on reef benthic evolution following a significant disturbance, modelled initial conditions for all simulations presented here represent the reef after a highly damaging event that has killed all corals and let the space be invaded by algal competitors (90% primary algal competitor, 10% secondary algal competitor). Thus, initial conditions are ephemeral and not representive of the steady state of the reef benthic ecosystem, the steady state being dependent on the type of simulated herbivore impact. For completeness, we have explored the sensitivity of the results presented in the following section to a range of initial conditions and have found that results are insensitive to wide variations in the ratio of primary to secondary algal competitors in the initial state (see electronic supplementary material, appendix S3).

**Figure 3.**
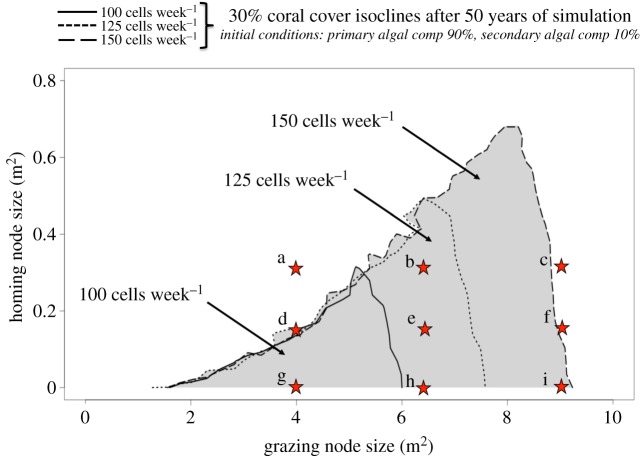
Isoclines of the mean coral cover after 50 years of simulation as a function of the homing node size (*Y*-axis) and the grazing node size (*X*-axis). From an initial substrate composed of 90% of primary algal competitor and 10% of secondary algal competitor, 30 simulations of 50 years have been run for a sample of 1200 grazing and homing node sizes. For each of 1200 pairs of values, the mean coral cover has been calculated over 30 simulations. The three isoclines represent the mean 30% coral cover threshold for three different values of *B*_M_, the maximal grazing impact. Inside the curves the coral cover is higher than 30% after 50 years (grey zone for *B*_M_ = 150 cells week^−1^), outside, it is lower. The area inside the curve is proportional to the diversity of space use leading to at least 30% coral cover after 50 years. The diversity of behaviours leading to at least 30% coral cover after 50 years is increasing with the value of the maximal grazing impact, opening towards ‘spatially less constrained’ behaviour (e.g. point c). The behaviours leading to a 30% coral cover after 50 years for the lower value of the maximal grazing impact are ‘spatially more constrained’ (e.g. point g).

Simulations were run over 50 years for a broad range of values for the grazing node size, homing node size and maximum grazing pressure (figure 3). By doing so, we have explored the impact of 1200 types of herbivore space use under three values of maximum grazing pressure: 100 cells week^−1^ (4% of the substrate per week), 125 cells week^−1^ (5% of the substrate per week) and 150 cells week^−1^ (6% of the substrate per week). Note that those values correspond to the foraging capacity of a mixed assemblage of coral reef herbivorous fishes at a biomass of around 5.5 g m^−2^, 7 g m^−2^ and 8.5 g m^−2^, respectively, or by 0.32 sea urchins m^−2^, 0.4 sea urchins m^−2^ and 0.48 sea urchins m^−2^, respectively [11,25]. One can also imagine, for example, a community defined by a density of sea urchins of 0.2 urchins m^−2^ and a herbivorous fishes biomass density of 3.5 g m^−2^ which will result in the model at the disappearance of 125 algal cells week^−1^, and so on. We chose to focus on these lower values of grazing intensity to highlight the role of spatial dynamics in reef recovery.

We define 30% coral cover after 50 years as a minimum coverage to qualify a reef substrate as recovered [26]. Note that qualitatively similar results were obtained with target values of coral cover ranging from 20% to 50%.

Using a Gaussian distribution to represent the grazing and homing behaviour led to qualitatively similar results as when using a uniform distribution. Because we focus on qualitative analyses, we will avoid redundancy by only presenting results obtained with a Gaussian distribution.

## 3. Results

**Figure 4.**
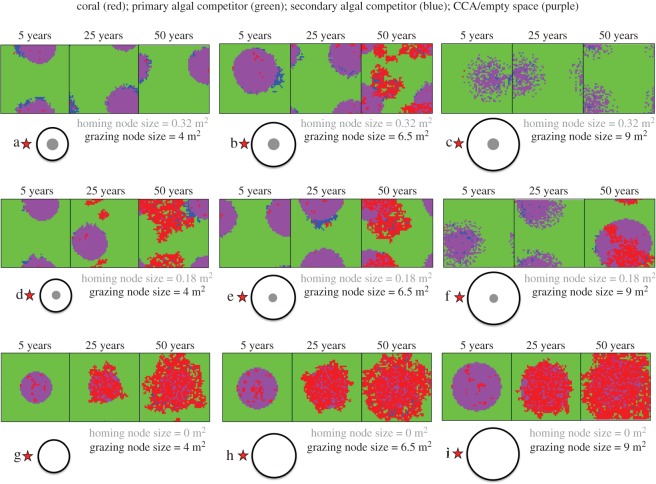
Representative snapshots of the modelled benthic landscapes. From an initial substrate composed of 90% of primary algal competitor and 10% of secondary algal competitor, nine simulations of 50 years have been run for a sample of nine grazing and homing node sizes with *B*_M_ = 150 cells week^−1^. For each of those nine pairs of values, three snapshots of the modelled 5 × 5 m benthic landscape have been taken after 5, 25 and 50 years of simulations, showing coverage by coral (red), secondary algal competitor (blue), first algal competitor (green) and CCA/empty space (purple). Each triplet of snapshots comes with scaled circles representing the homing (grey) and grazing (white) node size. The letter designation along with the red star sign indicates the position of the parameter values used to create figure 3.

To illustrate our results, we present in figure 4 snapshots of reef configuration for a range of space use parameters. Those snapshots offer an illustration of both the typical resulting benthic community and its spatial patterning due to the impact of space use patterns by herbivores.

Not all tested herbivore space use leads to a post-disturbance recovery within 50 years. In figure 3, the three curves represent the isocline for 30% coral cover after 50 years for three values of maximum bites per week: 100, 125 and 150 cells week^−1^. Inside the curve the coral cover is higher than 30% after 50 years, outside, it is lower. The area inside the curve is proportional to the diversity of space use patterns by the herbivore assemblage leading to at least 30% coral cover after 50 years. Indeed, as every point on the *homing node size* × *grazing node size* plane represents a given type of space use behaviour, a subset of the plane corresponds to a subset of space use behaviours. The larger this subset is, the higher the diversity of space use behaviours (i.e. the number of different types of space use behaviour) that lead to recovery of target levels of coral cover. The diversity of space use behaviour leading to at least 30% coral cover after 50 years is significantly different for the three maximum numbers of bites chosen in our simulations. Simulations with a maximum number of bites of 150 cells week^−1^ result in five of the nine types of space use leading to more than 30% coral cover after 50 years (marked by red stars in figure 3), while this number drops to only one in nine with 100 cells week^−1^ as the maximum bite rate.

Herbivores that are more ‘spatially constrained’ allow the reef to recover with a lower grazing pressure. If the maximum number of bites per week were to decrease (e.g. due to removal of herbivores by fishing) there exists a broadened range of movement behaviours for which the reef will no longer recover. For example, with a decrease in herbivory rate from 150 cells week^−1^ to 100 cells week^−1^, a collection of ‘less spatially constrained’ behaviours would no longer lead to recovery (see point h in figure 3). No such loss of recovery potential is observed for a comparable reduction in herbivory among ‘more spatially constrained’ behaviours (see point g in figure 3).

When comparing figures 3 and 4, we see that all recovering reefs (points b, e, g, h, i in both figures) are associated with a unique pattern in the benthic environment (figure 4), a pattern that we refer to as ‘halos’. A halo consists of a spatially autocorrelated change in the benthic cover. Inside the halo, all cells are made of the secondary algal competitor or crustose coralline algae/empty space whereas outside the primary algal competitor dominates.

**Figure 5.**
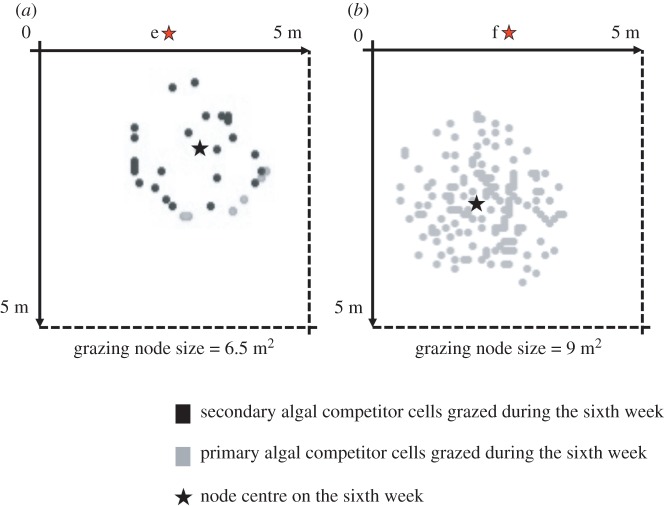
Composition of grazed cells for two grazing node sizes: (*a*) 6.5 m^2^ and (*b*) 9 m^2^. For both those values and over the 5 × 5 m grid, the algal cell grazed on the sixth week are denoted in black if it is a secondary algal competitor cell, in grey if it is a primary algal competitor cell, the grazing node centre being marked by a black star. With a 6.5 m^2^ grazing territory, the bites made on secondary algal competitor cells dominate and the total number of bites is lower compared with what is observed with a 9 m^2^, where no bites are made on the secondary algal competitor.

To understand how halos are created, we have monitored the nature and the localization of bites made on the algal community for two types of space use behaviour, types e and f*,* and provide a snapshot of those bites at model week 6 (figure 5). During the first couple of weeks both the 6.5 m^2^ (point e) and 9 m^2^ (point f) grazing nodes lead to major reductions of the primary algal competitor. However, after a given period of time, the more ‘spatially constrained’ type, e, then creates a halo in the primary algal competitor community, with herbivores subsequently shifting their benthic neighbourhood to newly recruited cover of the secondary algal competitor. Even if the secondary algal competitor recruits and grows rapidly, the rapid, repeated consumption forbids the recruitment of the primary algal competitor. Thus, the only algal cells left within the grazing area are secondary algal competitors. Note that as the halo was created within the first six weeks with ‘node e’ spatial grazing, it took more than 25 years for it to appear with node f (figures 4 and 5). Such a delay is due to the details of spatial configuration: a certain spatial stability is required to create a halo because they only appear once every algal cell in the grazing node has been consumed within a week. In other words halos appear only when the number of grazed cells equals or exceeds the number of algal cells present in the grazing node.

Benthic halos can be seen in every snapshot of figure 4, except for type c and the first 25 years of the simulation using type f*.* Even if a halo is created, spatial grazing according to type a does not lead to meaningful coral development (figures 3 and 4) as young coral recruits, without any help from the herbivores, almost immediately lose the competition for space with the primary algal competitor. However, the space use described by point d, which has the same grazing node size but a smaller homing node size, leads to a significant coral cover as its stability provides young corals enough time to reach the size at which they can compete with the primary algal competitor (900 cm^2^ in our model, see table 1).

Thus, the presence of a halo is a necessary but not sufficient condition for reef recovery. If a halo appears with the types a, d and f*,* those types of herbivore space use do not lead to appreciable reef recovery (as defined above).

**Figure 6.**
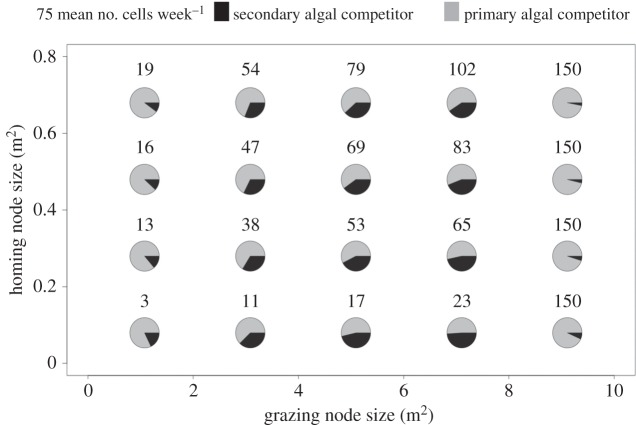
Mean diet of the herbivore community over 50 years as a function of the homing node size (*Y*-axis) and the grazing node size (*X*-axis). From an initial substrate composed of 90% of primary algal competitor and 10% of secondary algal competitor, 30 simulations of 50 years have been run for a sample of 20 couples of different grazing and homing node sizes. The two-colour pies represent the mean diet of the herbivores over 50 years (mean over 30 samples) with in black the secondary algal competitor ratio, in grey, the primary algal competitor one with underneath each pie the mean number of cells grazed over 50 years (mean over 30 samples with a maximum of 150 cells week^−1^). Two kinds of diet appear: (i) one for which the secondary algal competitor counts for more than 10% and up to 50% of the diet (when the grazing node size is lower than 8 m^2^); (ii) one for which the secondary algal competitor counts for less than 10% of the diet (when the grazing node size is higher than 8 m^2^). The mean number of cells grazed over 50 years increases with both the homing and grazing node size.

Based upon the foraging patterns of herbivores, two distinct types of local food availability emerge in our model for the range of grazing node types (figure 6). When the grazing node size exceeds 8 m^2^, the relative consumption of the secondary algal competitor reaches only 5% while the maximum number of bites, *B*_M_ = 150 cells week^−1^, is reached at the lowest value of the homing node size. However, when the grazing node is less than 8 m^2^, the relative consumption of the secondary algal competitor increases with the grazing node size while the total realized number of bites increases with the homing node size. As such, spatial patterns of herbivore movement are linked to the intensity of competition for food among herbivores when movements are more constrained (grazing node less than 8 m^2^; figure 6).

## 4. Discussion

An expansive literature has explored the variety of behaviours of herbivores on coral reefs, ranging from treatments of species-specific foraging specialization [27], context-specific foraging decisions [28] and responses to heterospecifics [29]. Importantly, most herbivore behavioural characteristics can be collapsed onto a common framework of resultant patterns of space use through time. The importance of spatial patterning in herbivory on emergent landscape dynamics has been highlighted in a number of general ecological studies [9,10], but has received only limited attention among coral reef studies. Investigating impacts of two extreme herbivore behaviours (i.e. idealized site-attached sea urchins versus spatially unconstrained herbivorous fishes), Sandin & McNamara [11] revealed that space use patterns can influence the long-term evolution of benthic patterning in a coral reef setting. However, the averaging typical of long-term estimates of reef development (on order of decades to centuries) is often insufficient for academic studies or resource management applications calling for views of short-term (i.e. year to decade) shifts in coral communities. Further, such extreme spatial representations fail to capture the variety of behaviours that are possible among reef herbivores. By exploring a continuum of representations for herbivore space use, this study provides generalized insights into how short- and longer term movement patterns of herbivores influence the development of reef benthic configuration. More specifically, with a focus on the rates of response in recently degraded reef habitats, we can advance our understanding of how patterns of space use by herbivores influence the potential for recovery of coral reef communities.

By focusing on patterns of space use among herbivores, we find that reef recovery is facilitated by a two-step process. First, herbivory opens space for corals to settle to the benthos. At the shortest timescales, herbivory has the capacity to remove algal competitors, especially primary algal competitors (i.e. late-successional algae with strong capacity for inter-specific competition for space), which commonly inhibit the settlement of early life-history corals through space pre-emption [6]. However, simple settlement of corals is insufficient to restore corals, as survivorship and competitive ability remains low until colonies reach sufficiently large sizes [30]. Thus, in a second step, the sustained and localized reduction of a primary algal competitor created by the herbivores is needed to facilitate the survival of these coral settlers by minimizing competition for space. More specifically, once a halo of competitor-free space is created, there emerges an ephemeral opportunity for early life-history corals to settle and perhaps recruit to the adult population. However, this competitor-free area needs to remain stable long enough to allow settling corals to grow to a size/stage where they develop the capacity to defend themselves from encroachment with spatial competitors. The presence of a halo for coral recruitment is thus necessary to obtain a recovered reef, but is not sufficient—longer term stability of this halo is also necessary.

The size and stability of patches available for coral settlement, recruitment and growth is intimately tied to the pattern of space use among herbivores. Let us take a given herbivore community which, daily, grazes within a given area (e.g. figure 5*a*). The size of that daily grazed area is small enough to lead to the removal of all algal cells, with the herbivore community feeding on freshly recruited secondary algal competitor cells, and this grazing pattern leads to the creation of a halo (figure 5*a*). In this context, it is possible for coral propagules to settle successfully. If that daily grazed area were larger, however, it would not be grazed as extensively of algal cells and algal succession would thus be permitted, profoundly reducing the probability of successful coral settlement (figure 5*b*). Thus, the grazing node size, reflecting shorter term pattern of movements, largely determines the creation of a halo, necessary for coral settlement.

However, coral settlement is only the first step towards reef recovery; growth is also necessary. To allow the growth of settlers, the daily grazed area that allowed their settlement needs to be stable in time, guaranteeing growth under conditions of limited competition with algae. The spatial stability of this grazed area depends on the homing node size, which reflects how herbivores move about their grazing territory on a longer term. If those longer term movements are small enough, recovery can happen (figures 3 and 4, point d); conditions for survival and growth of early life-history corals are maintained for a sufficiently long time interval. But if those longer term movements are too large, recovery is impossible despite the presence of settlers (figures 3 and 4, point a). Under this latter scenario, the conditions that facilitated settlement are spatially ephemeral and may not persist for enough time to enable early life-history corals to recruit to the adult population. Thus, if shorter term movements largely determine the creation of a halo (mandatory to facilitate coral recruitment), longer term movement determines its stability and thus coral growth capability. Consequently, only assessment of both types of movement allows for an understanding of the recovery capability provided by a given assemblage of herbivores.

Model results suggest that constrained patterning of herbivory may result in a systematic shift of the local benthic configuration, perhaps resulting in a change in the types of food ultimately available for the herbivore. Indeed, the space use behaviour leading to the creation of a halo can only, by definition, depend on secondary algal competitor recruits (e.g. young turf algae) within the grazing area. Specifically, the results of our model considering food availability for herbivores shows that spatially constrained herbivores can forage in such a way as to create a change in the benthos, consistently shifting small areas of primary algal competitor to areas of secondary algal competitor algae (figure 5). Those results point to a link between the successional rank of the primary producer being consumed and the size of the territory foraged by a grazer. The smaller a grazing territory is, the faster the grazing turnover of a given area will be, thus maintaining a foraging area in the early stages of algal succession. In cases where the herbivore maintains such a spatially constrained pattern of foraging regardless of associated benthic shifts, the conditions may exist for dietary specialization to evolve through natural selection. For example, the farming behaviour and specialized physiology exhibited by some species of pomacentrids may have been selectively favoured given the changes in benthic cover associated with spatially limited grazing patterns of the taxa (for more information about pomacentrid evolution, see Frédérich *et al*. [31]).

Thus, size and stability of a given grazing node both appear to be responsible for specific outcomes of a recovering reef as well as resulting diets for given taxa. Interestingly, the size and the stability of a given grazing node may depend not only on the topological nature of the reef, but also on both intra- and inter-specific interactions. Hence, we posit that the non-lethal effect of prey–predator interactions, as described by Lima [32], could also play a role here. For example, because of predator-induced changes in the prey behaviour, predators may have a cascading ecological impact beyond the actual act of predation [29,33–35]. If the presence of high numbers of predators were to lead to behavioural shifts in prey to avoid predation, possibly realized as a reduction in territory size, then a decrease in the predator population may lead the grazers to increase their territory size and thus reduce the spatial constraints on their grazing activity. Our results suggest that such behavioural changes that reduce spatial constraints of herbivores, even without changes in total algal consumption, can shift benthic dynamics in quantitative and noticeable ways (figure 3). As such, the depletion of predator populations has potential to slow coral recovery on damaged reefs.

The herbivory model presented in this work is simplified in an effort to explore model dynamics. It is critical, however, to consider how the models' simplifications capture the major dynamics of a notoriously complex community that is the coral reef. Firstly, our parametrization of bite behaviour does not reflect the bite of an individual herbivore, but instead a realized ‘rain of bites’ of a herbivore assemblage [19]. The impact of herbivores on coral reefs have traditionally been studied by quantifying relative feeding rates (number of bites per time point) on benthic algae [36,37]. The size of those bites is highly variable as it depends on the size and grazing behaviour of the observed grazers. However, in our model we represent grazing activity by the disappearance of a 100 cm^2^ algal cells and their transition towards crustose coralline algae/empty space. Again, such an apparent mismatch is explained by the fact that we have focused on grazing impacts at the spatial scale appropriate to the dynamics of spatial competition between coral and algae [11], which is in essence the summation and characterization of countless individual bites.

Secondly, we have simulated herbivore movement by scaling up from an individual. Within both the grazing and homing node distributions, the simulated movement of our general grazer is random. However, herbivores define their movements based on myriad factors including diet preference and territoriality [38]. Importantly, the behaviour of herbivores as modelled here includes non-random movement within defined areas, with herbivores finding and consuming only algal cells. Diet-specific movement behaviour, more complex than the one modelled in this work (e.g. with more specific discrimination of algal types), could also have an impact on the reef substrate dynamics. However, the results presented in this study appear to be qualitatively similar when food preference profiles are implemented into the model (see electronic supplementary material, appendix S1); the spatial patterns of herbivory appear to have a quantitatively dominant effect on resulting spatial patterns on the benthos. As such, the inclusion of more taxonomically refined data, i.e. more benthic groups, appears not to reduce the importance of spatial constraints in affecting benthic configurations and pattern evolution [9].

Finally, as we did not represent explicitly a collection of individual herbivores, the simulated number of cells grazed on the reef should not be seen as a simple proxy for herbivore density or biomass, but rather as the product of both the herbivore community composition and their individual-specific foraging behaviour. For example, a single spatial grazing type could reflect a large parrotfish or approximately 20 small urchins during the same period. Further, we emphasize that reference to the primary and secondary algal competitor using the terms macroalgae/thick turf and turf, respectively, should be considered carefully. The model presented here represents two groups of algae that differ in their competitive and succession rank. Thus, any direct link with a specific coral reef should be built on the identification of the algae fulfilling the model parametrizations rather than attempting a classification made only on physiological traits.

Our study offers a modelling framework designed to inform our understanding of reef ecology as well as considering reef conservation policy. Our results show that not all herbivore behaviour is equal in terms of providing recovery capabilities to reefs. We suggest future data collection efforts should provide species-specific values for sizes of grazing nodes and homing nodes, allowing for a site-specific assessment of the recovery capabilities of the herbivore community. A model that is parametrized to specific reef characteristics and conditions could have the potential to offer operational guidance for reef managers by more precisely evaluating the benefits of alternative scenarios of herbivore management. We hope that the model presented in this study will spawn field efforts in herbivore space use characterization that help to better allocate resources for species protection based upon species' subsequent impact on coral reef recovery and conservation.

## Supplementary Material

appendix 1

## Supplementary Material

appendix 2

## Supplementary Material

appendix 3
