# Supplementary material for: Herbivore space use influences coral reef recovery
Source: R Soc Open Sci. 2016 Jun 29;3(6):160262. doi: 10.1098/rsos.160262 (PMC4929919; doi:10.1098/rsos.160262)
Supplement: appendix 1 [file rsos160262supp1.pdf]

Article Type: Research article

Herbivore space use influences coral reef recovery: appendix 1

Yoan Eynaud[1*], Dylan E. McNamara[2], Stuart A. Sandin[1]

[1] *Center for Marine Biodiversity and Conservation, Scripps Institution of Oceanography, 9500 Gilman Drive, La Jolla, CA 92093-0202, USA*

[2] *Department of Physics and Physical Oceanography/Center for Marine Science, University of North Carolina, Wilmington, 601 South College Road, Wilmington, NC 28403, USA*

[*]*corresponding author, email: yeynaud@ucsd.edu*

Phone: +1 (858) 405-6296

Fax: +1 (858) 822-1267

In the model presented in this study, the probability for an algal cell to be grazed is independent of its type (i.e., primary algal competitor or secondary algal competitor). To understand the implication of such an assumption we have implemented two parameters that control for food preferences, P_{SAC} and P_{PAC}. P_{SAC} is the proportional preference for foraging on the secondary algal competitor, P_{SAC} is the proportional preference for foraging on the primary algal competitor, and the two parameters are linked such that $P_{SAC} + P_{PAC} = 1$.

The probability for an algal cell to be grazed depends on its type and is defined as, if the state of the cell is secondary algal competitor:

$P_{G,S}=P_G*P_{SAC,}$

or

$P_{G,P}=P_G*P_{PAC}$

if the state of the cell is primary algal competitor. P_G is the probability that a given algal cell will be grazed, a value that is drawn from the chosen distribution that define the grazing node (Gaussian of uniform). Using Gaussian distributions, the same pattern than the one presented in Fig.3 has been obtained for each of the following four profiles of relative preference for algal types: [P_{PAC}=0.25:P_{SAC}=0.75], [P_{PAC}=0.5:P_{SAC}=0.5], [P_{PAC}=0.75:P_{SAC}=0.25], [P_{PAC}=1:P_{SAC}=0]. Note that the profiles range from preference for secondary algal competitors, no preference, preference for primary algal competitors, and strong preference for primary algal competitors, respectively.

The differences among the four food preference profiles are shown for B_M=150 in Fig. A. In general, the results are qualitatively similar across all preference profiles, suggesting that the patterns of space use among herbivores strongly influence the results despite the specifics of algal food preference. Quantitatively, we see some distinctions among preference profiles. When $P_{SAC}>0$ the diversity of space use leading to at least 30 % coral cover after 50 years is increasing with the value of P_{SAC}. Also we note that in the scenario when the secondary algal competitor is never consumed (P_{SAC}=0), the diversity of space use leading to at least 30 % coral cover

after 50 years includes more space use types with large grazing node size but less space use type with large homing node size.

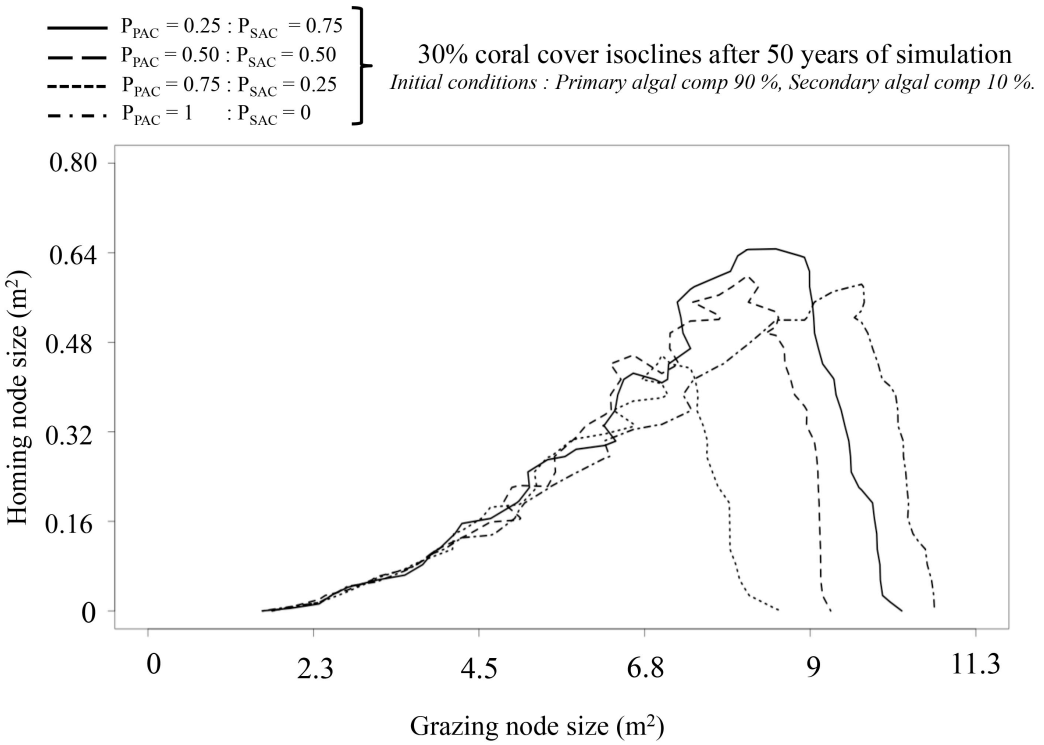

Figure A: Isoclines of the mean coral cover after 50 years of simulation as a function of the homing node size (Y-axis) and the grazing node size (X-axis). From an initial substrate composed of 90 % of primary algal competitor and 10 % of secondary algal competitor, 30 simulations of 50 years have been for a sample of 900 (30*30) grazing and homing node sizes. For each of 900 pairs of values, the mean coral cover has been calculated over 30 simulations. The four isoclines represent the mean 30 % coral cover threshold for 4 different food preference profiles: [P_{PAC}=0.25:P_{SAC}=0.75], [P_{PAC}=0.5:P_{SAC}=0.5], [P_{PAC}=0.75:P_{SAC}=0.25], [P_{PAC}=1:P_{SAC}=0]. Inside the curves the coral cover is higher than 30 % after 50 years. The area inside the curve is proportional to the diversity of space use leading to at least 30 % coral cover after 50 years. When $P_{SAC}>0$, the diversity of space use leading to at least 30 % coral cover after 50 years is increasing with the value of P_{SAC}. When the secondary algal competitor is never consumed (P_{SAC}=0), the diversity of space use leading to at least 30 % coral cover after 50 years comparatively include more space use type with large grazing node size but less space use type with large homing node size.
