# Supplementary material for: Herbivore space use influences coral reef recovery
Source: R Soc Open Sci. 2016 Jun 29;3(6):160262. doi: 10.1098/rsos.160262 (PMC4929919; doi:10.1098/rsos.160262)
Supplement: appendix 2 [file rsos160262supp2.pdf]

Article Type: Research article

Herbivore space use influences coral reef recovery: appendix 2

Yoan Eynaud[1*], Dylan E. McNamara[2], Stuart A. Sandin[1]

[1] *Center for Marine Biodiversity and Conservation, Scripps Institution of Oceanography, 9500 Gilman Drive, La Jolla, CA 92093-0202, USA*

[2] *Department of Physics and Physical Oceanography/Center for Marine Science, University of North Carolina, Wilmington, 601 South College Road, Wilmington, NC 28403, USA*

[*] *corresponding author, email: yeynaud@ucsd.edu*

Phone: +1 (858) 405-6296

Fax: +1 (858) 822-1267

The Gaussian distribution

The topological structure of our modeled benthos, a torus, forbids us to use a Gaussian distribution *sensu stricto.* Indeed, a Gaussian distribution, *sensu stricto,* is defined on an infinite domain, whereas a torus is a finite domain. Thus, we use circularly truncated distributions for both the homing and grazing node. The

truncation will take place at a distance of two standard deviations from the node center, which encompasses 95.4 % of the complete Gaussian distribution. The radius of the grazing node (R_G) as well as the homing one (R_H) are then defined as:

$$R_G = 2 * \sqrt{V_G}$$

$$R_H = 2 * \sqrt{V_H}$$

Using a Gaussian distribution, our grazing node is defined as follow: from a certain position on a 2-dimensions domain, the probability for a cell to be grazed decreases, in every direction, from a maximum value following a Gaussian law with a variance equals to V_G and equals 0 if this distance is superior to R_G.

At each time step, the node center may be relocated in another cell. Again, from the previous position of the node center on the 2-dimensions domain, the probability for a cell to shelter it next times decreased, in every direction, from a maximum value following a Gaussian law with a variance equals to V_H and equals 0 if this distance is superior to R_H.

For parameter units and descriptions, see table 1.

The Uniform distribution

Using a uniform distribution, the probability for an algal cell to be grazed each week is here equal to:

$$P_G = \frac{1}{n_G}$$

where n_G is the number of algal cells (i.e. turf and macroalgae) in the grazing node of radius R_G. The probability for a cell to become the next node center is here equal to:

$$P_H = \frac{1}{n_H}$$

where n_H is the number of cell in the circle of diameter R_H .

Table 1: Parameter definition for simulation model.

Parameters	*Interpretation*	*Range*	*Unit*
V_G	Grazing movement variance	0 - 15000	cm^2
V_H	Homing movement variance	0-500	cm^2
n_G	Number of algal cells in the grazing node	0-1000	-
n_H	Number of cells in the homing node	0-80	-
P_H	Probability to be the next node center	0-1	-
P_G	Probability to be grazed	0-1	-

R_G	Radius of the grazing node	0-250	cm
R_H	Radius of the homing node	0-50	cm
B_M	Maximum grazing impact	100/125/150	cells.week^{-1}
