# Supplementary material for: Herbivore space use influences coral reef recovery
Source: R Soc Open Sci. 2016 Jun 29;3(6):160262. doi: 10.1098/rsos.160262 (PMC4929919; doi:10.1098/rsos.160262)
Supplement: appendix 3 [file rsos160262supp3.pdf]

Article Type: Research article

Herbivore space use influences coral reef recovery: appendix 3

Yoan Eynaud[1*], Dylan E. McNamara[2], Stuart A. Sandin[1]

[1] *Center for Marine Biodiversity and Conservation, Scripps Institution of Oceanography, 9500 Gilman Drive, La Jolla, CA 92093-0202, USA*

[2] *Department of Physics and Physical Oceanography/Center for Marine Science, University of North Carolina, Wilmington, 601 South College Road, Wilmington, NC 28403, USA*

[*]*corresponding author, email: yeynaud@ucsd.edu*

Phone: +1 (858) 405-6296

Fax: +1 (858) 822-1267

In all the simulations presented in this study, the initial conditions were set as follow: 90% primary algal competitor, 10% secondary algal competitor with the goal to simulate a post-disturbance state. In some systems, the observed dynamic depends highly on the initial conditions. Thus, it is necessary to explore how the initial conditions influence the output of the model.

Here, we have chosen to explore the output of three sets of initial conditions: (i) 100% CCA/empty space; (ii) 100% secondary algal competitor; (iii) 100% primary algal competitor. In the Fig. A, one can see that the outputs obtained with the three different sets of initial conditions are identical after 300 days. Hence, the results presented in this study are similar for wide variations in the ratio of primary to secondary algal competitor.

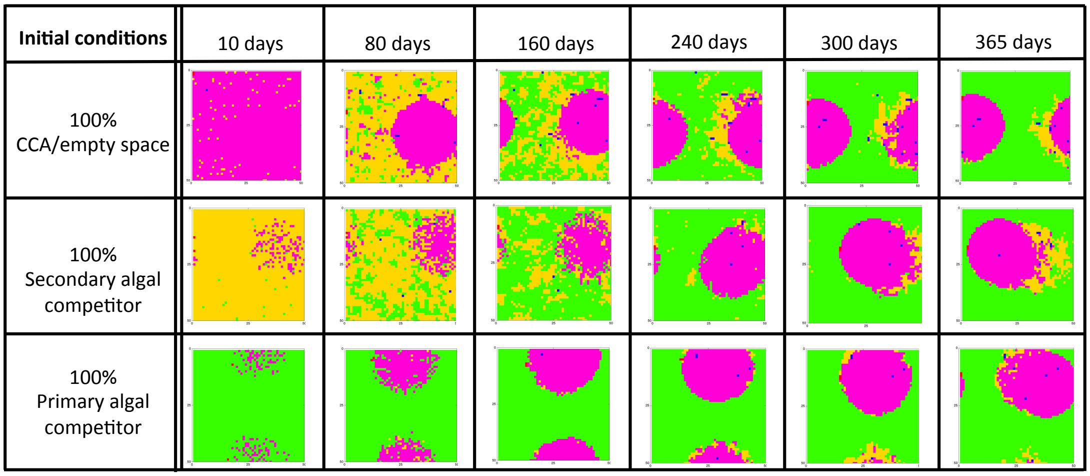

Figure A: Influence of the initial conditions. For each set of initial conditions: (i) 100% CCA/empty space, (ii) 100% secondary algal competitor and (iii) 100% primary algal competitor, we present a snapshots of the modeled 5x5 m benthic landscape after 10, 80, 160, 240, 300 and 365 days of simulations, showing coverage by coral (red), secondary algal competitor (blue), first algal competitor (green) and CCA/empty space (purple). Here the herbivore space use is defined as follow: grazing node size= 5 m^2, homing node size = 0.1 m^2.
